# Mortality after surgery for primary hyperparathyroidism: results from a nationwide cohort

**DOI:** 10.1093/bjs/znab017

**Published:** 2021-04-11

**Authors:** M Nilsson, K Ivarsson, M Thier, E Nordenström, A Bergenfelz, M Almquist

**Affiliations:** 1 Department of Surgery, Skåne University Hospital, Lund, Sweden; 2 Department of Clinical Sciences, Lund University, Lund, Sweden; 3 Department of Child and Adolescent Psychiatry, Psychiatry, Skåne, Sweden

## Abstract

**Background:**

Contemporary patients with primary hyperparathyroidism are often diagnosed with mildly raised serum calcium levels. Previous studies have reported increased mortality in patients with primary hyperparathyroidism. This retrospective cohort study aimed to examine whether contemporary patients operated for primary hyperparathyroidism have higher mortality than the general population, and whether mortality in these patients is associated with serum calcium concentration, adenoma weight or multiglandular disease.

**Methods:**

Patients from a Swedish national cohort consisting of patients registered in the Scandinavian Quality Register for Thyroid, Parathyroid, and Adrenal Surgery 2003–2013, were matched with population controls. The National Patient Register, the Swedish Cause of Death Register, and socioeconomic data were cross-linked. End of follow-up was 10 years after surgery, 31 December 2015, or emigration. Mortality was analysed by standardized mortality ratio, Kaplan–Meier survival estimates, and univariable and multivariable Cox regression. Multiple imputation by chained equations was performed on missing data.

**Results:**

After exclusions, there were 5009 patients with primary hyperparathyroidism and 14 983 controls. Multivariable Cox regression analysis adjusted for age, sex, Charlson Co-morbidity Index, marital status, level of education, disposable income, and period of surgery showed lower mortality in patients than controls (hazard ratio (HR) 0.83, 95 per cent c.i. 0.75 to 0.92). In univariable Cox regression of mortality in patients, serum calcium concentration (mmoles per litre) was associated with mortality (HR 2.20, 1.53 to 3.16). This association remained in multivariable Cox regression after multiple imputation (HR 1.79, 1.19 to 2.70).

**Conclusion:**

Mortality was not increased in patients operated for primary hyperparathyroidism compared with controls in a contemporary setting. Preoperative serum calcium concentration might, however, influence survival.

## Teaser

In this cohort study of patients who underwent parathyroidectomy for primary hyperparathyroidism in a contemporary setting, in which a national cohort of 5009 patients was matched with population controls, mortality was not increased for patients compared with controls. However, preoperative serum calcium level was associated with mortality, and might therefore be useful in patient selection for surgery.

## Introduction

Parathyroidectomy for primary hyperparathyroidism (pHPT) is the second most common procedure in endocrine surgery. In 2018, parathyroidectomy was performed in 15.2 per 100 000 inhabitants in Sweden^1^^,^^2^. Since the introduction of automated laboratory determination of serum calcium concentration, there has been a shift toward treating patients with lower levels of serum calcium, parathyroid hormone (PTH), and lower adenoma weight, and also patients with fewer complications from the disease at the time of surgery^3–12^.

Several previous studies^13–17^ have demonstrated excess mortality in patients with pHPT compared with the general population, probably related to manifest preoperative complications of the disease. More recent studies^18–20^ have, however, not supported this finding, and it is unclear whether surgery for pHPT improves survival compared with conservative management^16^^,^^21^. More severe pHPT with higher serum calcium and PTH levels, and larger adenomas, might be associated with increased mortality even after surgery^18^^,^^22^.

Reflecting the lack of clear evidence, the present guidelines give differing advice regarding the indications for surgery in patients with pHPT^23–25^. There is consensus that patients with classical complications of pHPT, such as nephrolithiasis, fragility fractures, and osteoporosis, markedly raised serum calcium level or age below 50 years should be considered for surgery. The American Association of Endocrine Surgeons guidelines also recommend surgery for patients with creatinine clearance below 60 ml/min, 24-h urinary calcium more than 400 mg (equal to 10 mmol) or non-specific symptoms such as fatigue, depression, anxiety, irritability, cognitive impairment, and sleep disorders.

This study aimed to examine whether there is excess mortality in patients operated for pHPT compared with population controls in a modern setting, and to assess whether mortality in patients who have surgery for pHPT is associated with preoperative serum calcium levels, adenoma weight or multiglandular disease.

## Methods

This study was carried out and reported according to the STROBE statement^26^. The study was approved by the local ethical committee at Lund University (2016/26).

### Study population

The cohort comprised patients operated for HPT and registered in the Scandinavian Quality Register for Thyroid, Parathyroid, and Adrenal Surgery (SQRTPA). Registration in the SQRTPA was piloted in 2003–2004, and was in full operation by 2005. At the end of the study period, SQRTPA had a coverage of 97 per cent, defined as the number of parathyroidectomies at participating centres as a proportion of the total number in Sweden^27^. This database contains information on preoperative characteristics, surgery, histology, and follow-up. Patients with a diagnosis of pHPT who underwent operation between 1 January 2003 and 31 December 2013 were included. Patients with hereditary HPT, parathyroid carcinoma on histology, and those who had been treated with lithium were not eligible for the study, even though they had been categorized as having pHPT at registration. Patients with HPT are either classified as having pHPT or secondary HPT in the SQRTPA. Patients with a diagnosis of secondary HPT were not eligible, and were not included in the data retrieval.

For each patient, three controls were matched with respect to sex, age, and municipality by Statistics Sweden. All controls were selected to be alive at the date of surgery, to avoid immortal time bias. Patients with an invalid or reused personal identification number, negative exploration, persistent disease defined as hypercalcaemia (ionized calcium over 1.33 mmol/l or total calcium above 2.50 mmol/l with albumin within the reference range) at the 6-month follow-up, reoperation or missing follow-up were excluded, along with their respective controls. Multiglandular disease was defined as more than one excised gland and primary histological diagnosis not being adenoma.

Socioeconomic data, information on hospital admissions, and time and causes of death for patients and their controls were retrieved by cross-linking with Statistics Sweden, the National Patient Register, and the Swedish Cause of Death Register held by the National Board of Health and Welfare. After linkage, controls who had undergone parathyroid surgery were also excluded.

To calculate the survival time, controls were assigned the date of surgery of their respective patient, hereafter referred to as *d*. The end of follow-up was 31 December 2015, 10 years after *d* or emigration, whichever came first. The Charlson Co-morbidity Index (CCI) score^28^^,^^29^ on *d* was calculated with hospital discharge diagnoses up until *d*, using the algorithm described by Quan and colleagues^30^.

### Statistical analysis

Descriptive statistics are presented as mean(s.d.) for normally distributed and median (i.q.r.) for skewed variables. Parametric and non-parametric tests were used as appropriate to test differences between groups. Standardized mortality ratios (SMRs) were estimated for patients with pHPT and controls in the cohort, for men and women separately, using mortality rates derived from the online database of Statistics Sweden in 5-year age bands for each year 2003–2015^31^^,^^32^.

Kaplan–Meier estimates, log rank tests, and Cox regression analyses were employed for univariable mortality analysis. Time of entry was the date of surgery or the corresponding date for controls (*d*); exit was death, emigration or end of follow-up. Statistical interaction was evaluated for socioeconomic and biochemical variables using Cox regression analyses and, when necessary, likelihood ratio tests. For multivariable mortality analysis of patients *versus* controls, a multivariable Cox regression model was fitted, adjusted for age over 65 years, sex, CCI score (0, 1, 2 or more), marital status, level of education, disposable income in quartiles, and time period of surgery (2003–2007, 2008–2010, 2011–2013).

To evaluate the risk of death among patients who had surgery for pHPT, multivariable Cox regression models adjusted for the same factors as above were fitted for preoperative total calcium, adenoma weight, and multiglandular disease, using data from the patient cohort only. Biochemical variables were evaluated both continuously and in categories. The proportional hazards assumption was assessed for each Cox regression visually and using Schoenfeld partial residuals^33^.

Patterns of missingness was evaluated visually and by logistic regression. Missing values were deemed likely to be missing at random, enabling multiple imputation by chained equations^34^. Preoperative total calcium and total calcium concentration at 6 months after surgery were included as linear predictors, and adenoma histology using a logistic regression model. Adenoma weight was log-transformed owing to skewness and imputed as a linear predictor conditionally on the finding of adenoma on histology. It was not possible to include multiglandular disease owing to collinearity with adenoma histology. Marital status and educational level were included using ordered logistic regression models. The survival outcome was included as the Nelson–Aalen estimator for time to death together with the censoring indicator (complete). Age, sex, disposable income, year of surgery, and CCI score were also included (complete). Altogether, 20 imputed data sets were created using 30 iterations. Distributions of imputed data were assessed visually for convergence. Cox regression was performed on each imputed data set and combined according to Rubin’s rules^35^.

Subgroup analyses were undertaken of complete cases and imputed data separately by sex, marital status, and categories of disposable income, and finally excluding controls without hospital admission before *d.* For the risk stratification model, additional subgroup analyses were performed separately on patients with uniglandular disease and adenoma on histology.

All tests were two-sided and *P* < 0.05 was considered significant. For all statistical analyses, Stata^®^ SE version 13.1 (StataCorp, College Station, Texas, USA) was employed.

## Results

**Fig. 1:**
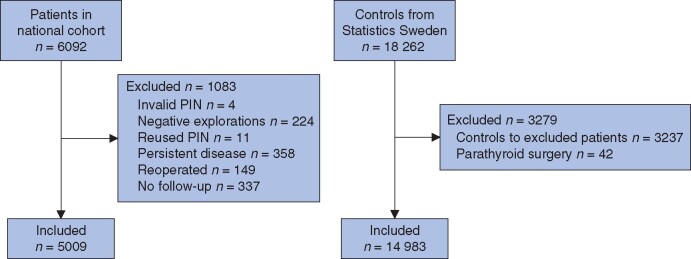
Study flow chart PIN, personal identification number.

After exclusions, 5009 patients and 14 983 controls remained in the national cohort (*Fig. 1*). Patients had 29 419 person-years at risk and controls had 87 493 person-years. Median follow-up was 5.7 (i.q.r. 3.8–7.9) years, and was the same for patients and controls. As national coverage of the SQRTPA increased gradually during the study period, only 8.2 per cent of patients completed 10 years of follow-up.

### Missing data

Some 27.8 per cent of patients had at least one variable of any of the multivariable Cox regression models missing. However, 97.4 per cent had complete information on all variables of the multivariable model with calcium as predictor. The largest fraction of missing information was 0.241 (for total calcium at 6 months).

### Preoperative and perioperative characteristics

Patients and controls were well matched, with similar distributions of socioeconomic factors such as marital status, disposable income, and educational level. However, the CCI score was significantly higher in patients (*Table 1*). Preoperative laboratory findings, symptoms, and complications related to pHPT as well as perioperative characteristics are summarized in *Table 2*.

**Table 1:** Demographics and co-morbidity

	Patients		Controls		***P*** ^‡^
	*n*	Value	*n*	Value	
**Age (years)** *	5009	61.7(13.7)	14 983	61.7(13.7)	0.903^§^
**Men**	5009	1129 (22.5)	14 983	3380 (22.6)	0.977
**Unmarried**	4982	2346 (47.1)	14 851	7053 (47.5)	0.623
**Charlson Co-Morbidity Index score^†^**	5009	0 (0–1)	14 983	0 (0–0)	< 0.001^¶^
**Disposable income (€)** ^†^	5009	16 525 (11 945–23 795)	14 981	16 420 (11 804–23 929)	0.250^¶^
**Elementary school only**	4955	1324 (26.7)	14 738	4304 (29.2)	0.003
**Year of surgery**	5009		14 983		0.999
2003–2007		1428 (28.5)		4272 (28.5)	
2008–2010		1756 (35.1)		5249 (35.0)	
2011–2013		1825 (36.4)		5462 (36.5)	

Values in parentheses are percentages unless indicated otherwise; values are *mean(s.d.) and † median (i.q.r.). ‡ χ^2^ test unless, except § *t* test and ¶ Wilcoxon rank-sum test.

**Table 2:** Preoperative and operative characteristics

	*n*	Value
Total calcium (mmol/l)*	4958	2.78(0.20)
Ionized calcium (mmol/l)*	4955	1.46(0.10)
Cognitive symptoms	148^‡^	148 (3.0)
Fatigue	501^‡^	501 (10.0)
Urinary stone	126^‡^	126 (2.5)
Skeletal disease	229^‡^	229 (4.6)
Unilateral or focused exploration	5009	2399 (47.9)
Duration of operation (min)^†^	3876	70 (45–103)
Multiglandular disease	4101	394 (9.6)
Adenoma on histological examination	4970	4359 (87.7)
Adenoma weight (g)^†^	3702	0.6 (0.3–1.2)

Values in parentheses are percentages unless indicated otherwise; values are *mean(s.d.) and ^†^median (i.q.r.). ^‡^Only registered with positive finding.

### Mortality

There were no substantial imbalances in causes of death between patients and controls (*Table S1*). The SMR was not increased for either men or women in the patient group, but was increased among female controls, compared with the general Swedish population (SMR 1.11, 95 per cent c.i. 1.05 to 1.17) (*Table S2*).

In univariable analysis of patients *versus* controls, age, CCI score, disposable income, marital status, and educational level were all associated with mortality (*Table S3*). In univariable analysis, patients who had surgery for pHPT did not have increased mortality compared with controls (*Fig. S1*).

**Fig. 2:**
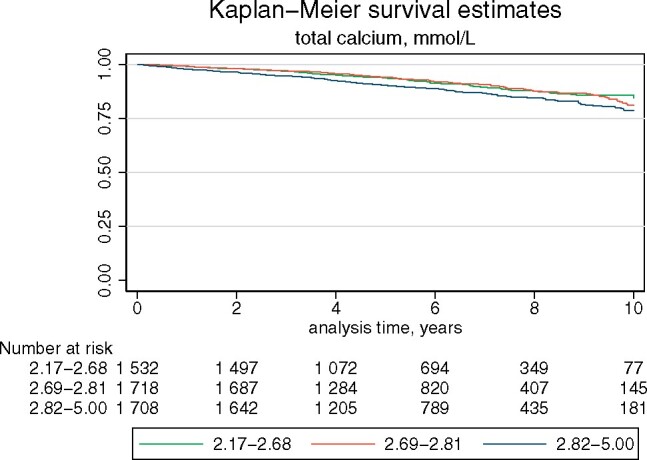
Kaplan–Meier survival estimates in relation to total preoperative calcium concentration *P* < 0.001 (log rank test ).

Among patients, total preoperative calcium concentration was associated with mortality in univariable Cox regression (hazard ratio (HR) 2.20, 95 per cent c.i. 1.53 to 3.16) (*Fig. 2* and *Table S4*). There was a trend toward an association between mortality and adenoma weight (*Fig. 2* and *Table S4*).

In multivariable Cox regression analysis, patients had better survival than controls (HR 0.83, 0.75 to 0.92) after adjustment for age, sex, CCI score, marital status, education level, disposable income, and period of surgery. The subgroup analyses showed a stronger effect of preoperative calcium in patients with income in the lowest quartile, uniglandular disease or adenoma on histology; detailed results are available in *Table S5*.

No statistical interactions were found that were necessary to include in the multiple imputation or statistical multivariable models.

In the multivariable models stratifying risk for patients, there was no significant association between preoperative calcium level, adenoma weight or multiglandular disease and mortality. In analyses of imputed data, however, the effect of calcium on mortality was statistically significant (*Table 3*).

**Table 3:** Analysis of main risk factors for death in multivariable Cox regression models

	Available cases		Imputed data	
	**Hazard ratio**	***P***	**Hazard ratio**	***P***
**Total calcium (mmol/l)**				
Continuous	1.49 (0.97, 2.30)	0.072	1.79 (1.19, 2.70)	0.005
2.17–2.68	1.00 (reference)		1.00 (reference)	
2.69–2.81	1.04 (0.82, 1.32)	0.731	1.02 (0.81, 1.29)	0.853
2.82–5.00	1.20 (0.96, 1.51)	0.108	1.30 (1.04, 1.62)	0.022
**Adenoma weight (g)**				
Continuous	1.00 (0.95, 1.05)	0.970	1.01 (0.97, 1.06)	0.531
0.05–0.37	1.00 (reference)		1.00 (reference)	
0.38–0.88	1.04 (0.79, 1.36)	0.785	1.11 (0.88, 1.41)	0.366
0.89–50.0	1.01 (0.77, 1.32)	0.930	1.07 (0.85, 1.35)	0.567
**Multiglandular disease**	1.18 (0.86, 1.62)	0.310	Not selected	

Values in parentheses are 95 per cent confidence intervals. Each model was adjusted for age over 65 years, sex, Charlson Co-morbidity Index score (0, 1, 2 or more), marital status, level of education, disposable income in quartiles, and period of surgery.

## Discussion

The main finding of the present study is that mortality in patients undergoing surgery for pHPT is not increased from that of matched controls from the Swedish population. This is in contrast to previous studies^13–17^ that reported increased mortality among patients with pHPT. Several studies^5^^,^^9^^,^^10^ have indicated that contemporary patients have milder disease, and thus fewer complications due to pHPT. It is possible that the previously described increased mortality was the result of more extensive end-organ damage from pHPT. The lack of excess mortality in patients could also be explained by patients being treated earlier in the course of pHPT, before end-organ damage had occurred. Furthermore, patients with biochemically persistent pHPT after surgery were excluded from the present study, in contrast to previous studies^5^^,^^13–18^^,^^20^^,^^21^^,^^36^^,^^37^ of mortality after surgery for pHPT. It is unclear whether the present results imply less benefit from surgery or, on the contrary, success of earlier surgical treatment, highlighting the need for either a randomized trial, or a large observational study with a non-surgical control group^6^^,^^12^^,^^19^^,^^20^.

In agreement with the Rochester cohort of biochemically screened patients^19^, mortality was significantly lower for patients in the national cohort than for their controls, despite correction for co-morbidities and socioeconomic factors. It is possible that treatment of other conditions detected in the evaluation for surgery influenced survival. There could also have been selection bias that was not taken into account; for example, patients seeking medical attention for symptoms and conditions related to pHPT could have been healthier or more healthcare-consuming. Although the CCI score detects many conditions that increase mortality risk, only discharge diagnoses are used, which potentially miss co-morbidities only registered in outpatient care, leading to underestimation of co-morbidities among controls. In addition, data were not available on other risk factors, such as smoking, alcohol use, and physical activity. It is possible that patients undergoing surgery were healthier in these respects, confounding the comparison between patients and controls.

In this national Swedish cohort, preoperative serum calcium level had a stronger association with mortality than adenoma weight or multiglandular disease. The association between serum calcium concentration and mortality is in line with previous findings^5^^,^^22^^,^^36^, and could hypothetically be explained by more pronounced end-organ damage with higher serum calcium. Therefore, serum calcium levels might be useful in patient selection for surgery, especially in the absence of classical complications such as urinary stone formation, osteoporosis, or severe hypercalcaemia.

Limitations of this study include factors related both to its design and the patient data. Included patients were operated, and had therefore been selected as being fit for surgery, and having complications or symptomatology necessitating intervention. This could, theoretically, both exaggerate and diminish the possible effect of surgery on mortality. Although adjustment was made for CCI score, and patients had more co-morbidities than controls, there might have been undetected disease in the controls. It was not possible to include patients who were managed conservatively in the investigation, as the cohort is based on a quality registry for endocrine surgery. The authors had no access to a separate non-surgical cohort of patients with pHPT. The laboratory data were collected over a long time span, and from a large number of different facilities. This means that the laboratory methods used to produce the data might have been inconsistent. It was not possible to validate the pHPT diagnosis owing to lack of biochemical data (only serum calcium level is registered in the SQRTPA). It would also not have been feasible to retrieve and scrutinize medical records of the complete cohort.

Within the context of these limitations, this investigation showed that mortality was not increased in patients operated for pHPT compared with controls. Preoperative serum calcium level was associated with mortality and might be useful in patient selection for surgery.

## Funding

Funding for this study was provided by the Thelma Zoégas Fond för Medicinsk forskning, Thorsten Birger Segerfalks Stiftelse, Carl J. Michaelsens Donationsfond, Anna-Lisa och Sven Eric Lundgrens stiftelse för medicinsk forskning, and Skånes Universitetssjukhus stiftelser och donationer.

## Supplementary Material

znab017_Supplementary_Data

## Notes

Presented to the Annual Meeting of the Swedish Surgical Society (Kirurgveckan), Norrköping, Sweden, August 2019
