# Supplementary material for: Mortality after surgery for primary hyperparathyroidism: results from a nationwide cohort
Source: Br J Surg. 2021 Apr 11;108(7):858–63. doi: 10.1093/bjs/znab017 (PMC10364903; doi:10.1093/bjs/znab017)
Supplement: znab017_Supplementary_Data [file znab017_supplementary_data.doc]

# Mortality After Surgery for Primary Hyperparathyroidism, Supplement

Martin Nilsson, Kerstin Ivarsson, Mark Thier, Erik Nordenström, Anders Bergenfelz, Martin Almquist

### Table S1. Causes of death

| **Disease group ICD10** | **Patients** | **Controls** |
| --- | --- | --- |
| Cardiovascular I00–I99 | 196 | 571 |
| Tumor C00–D48 | 162 | 502 |
| Respiratory J00–J99 | 23 | 97 |
| Endocrine E00–E90 | 13 | 42 |
| Urogenital N00–N99 | 9 | 19 |
| Gastrointestinal K00–K93 | 1 | 6 |
| Psychiatric F00–F99 | 21 | 94 |
| Neurologic G00–G99 | 20 | 84 |
| Trauma, violence V01–Y99 | 24 | 65 |
| Infectious A00–B99 | 11 | 39 |
| Miscellaneous | 23 | 109 |
| Total | 503 | 1 628 |

### Table S2. Standardized mortality ratio

|  | **Men** | | **Women** | |
| --- | --- | --- | --- | --- |
|  | **Patients** | **Controls** | **Patients** | **Controls** |
| Deceased | 137 | 427 | 365 | 1 199 |
| Expected | 132.8 | 400.0 | 372.3 | 1 080.8 |
| SMR (CI 95%) | 1.03 (0.87–1.22) | 1.07 (0.97–1.17) | 0.98 (0.88–1.09) | 1.11 (1.05–1.17) |

### Table S3. Univariable Cox-regression, patients and controls

|  | **Available cases** | | **Complete cases** (N=4 929+14 613) | |
| --- | --- | --- | --- | --- |
|  | **HR (CI 95%)** | ***p*** | **HR (CI 95%)** | ***p*** |
| Patient | 0.92 (0.83–1.01) | 0.084 | 0.94 (0.85–1.05) | 0.282 |
| Age  continuous, years  <65 years  >65 years | 1.11 (1.11–1.12)  1.00 (ref)  7.52 (4.52–12.5) | <0.001  <0.001 | 1.12 (1.11–1.12)  1.00 (ref)  7.34 (6.53–8.25) | <0.001  <0.001 |
| Sex  female  male | 1.00 (ref)  1.26 (1.14–1.38) | <0.001 | 1.00 (ref)  1.26 (1.14–1.40) | <0.001 |
| Charlson’s score  continuous, p  0  1  ≥2 | 1.59 (1.56–1.63)  1.00 (ref)  3.91 (3.55–4.30)  9.60 (8.56–10.8) | <0.001  <0.001  <0.001 | 1.58 (1.54–1.61)  1.00 (ref)  3.82 (3.46–4.22)  8.96 (7.93–10.13) | <0.001  <0.001  <0.001 |
| Disposable income (SEK)  q1 (–36,079–)  q2 (120,261–)  q3 (167,058–)  q4 (242,669–) | 5.07 (4.29–5.99)  4.27 (3.60–5.06)  1.48 (1.22–1.80)  1.00 (ref) | <0.001  <0.001  <0.001 | 4.97 (4.17–5.93)  4.48 (3.75–5.35)  1.55 (1.27–1.90)  1.00 (ref) | <0.001  <0.001  <0.001 |
| Civil status  married  unmarried | 1.00 (ref)  1.77 (1.62–1.93) | <0.001 | 1.76 (1.61–1.93) | <0.001 |
| Educational level  elementary school  upper secondary school  higher education | 3.67 (3.23–4.18)  1.66 (1.45–1.90)  1.00 (ref) | <0.001  <0.001 | 3.65 (3.20–4.17)  1.68 (1.46–1.93)  1.00 (ref) | <0.001  <0.001 |
| Year of surgery  2003–  2008–  2011– | 1.00 (ref)  0.84 (0.76–0.93)  0.75 (0.65–0.86) | 0.001  <0.001 | 1.00 (ref)  0.87 (0.78–0.96)  0.74 (0.64–0.86) | 0.008  <0.001 |

### Table S4. Univariable Cox-regression before (complete cases) and after multiple imputation, patients

|  | **Available cases** | | **Complete cases** (N = 3 126) | | **Imputed data** | |
| --- | --- | --- | --- | --- | --- | --- |
|  | **HR (CI 95%)** | ***p*** | **HR (CI 95%)** | ***p*** | **HR (CI 95%)** | ***p*** |
| Total calcium  continuous, mmol/L  2.17–  2.69–  2.82– | 2.20 (1.53–3.16)  1.00 (ref)  1.00 (0.79–1.26)  1.40 (1.13–1.75) | <0.001  0.967  0.002 | 2.30 (1.35–3.91)  1.00 (ref)  0.90 (0.65–1.24)  1.30 (0.97–1.75) | 0.002  0.508  0.079 | 2.20 (1.53–3.17)  1.00 (ref)  0.98 (0.78–1.24)  1.46 (1.17–1.82) | <0.001  0.889  0.001 |
| Adenoma weight  continuous, g  0.05–  0.38–  0.89– | 1.00 (0.96–1.04)  1.00 (ref)  1.21 (0.93–1.57)  1.26 (0.97–1.63) | 0.977  0.160  0.078 | 1.00 (0.96–1.05)  1.00 (ref)  1.06 (0.79–1.43)  1.10 (0.82–1.48) | 0.987  0.680  0.518 | 1.01 (0.97–1.04)  1.00 (ref)  1.18 (0.94–1.49)  1.23 (0.98–1.53) | 0.781  0.157  0.069 |
| Multiglandular disease | 1.16 (0.85–1.57) | 0.344 | collinearity |  | collinearity |  |

### Tables S5a–j. Subgroup analyses. Main exposures of risk stratification multivariable Cox regression models. Each model adjusted for age over 65 years, sex, Charlson’s comorbidity score (0, 1, ≥2 points), marital status, level of education, disposable income in quartiles and period of surgery.

| **5a** | **Available cases** | | **Imputed data** | |
| --- | --- | --- | --- | --- |
| ***Men*** | **HR (CI 95%)** | ***p*** | **HR (CI 95%)** | ***p*** |
| Total calcium  continuous, mmol/L  2.17–  2.69–  2.82– | 1.54 (0.68–3.50)  1.00 (ref)  1.18 0.71–1.98)  1.44 (0.91–2.28) | 0.304  0.520  0.116 | 1.65 (0.75–3.64)  1.00 (ref)  1.25 (0.77–2.03)  1.47 (0.94–2.31) | 0.217  0.369  0.095 |
| Adenoma weight  continuous, g  0.05–  0.38–  0.89– | 0.98 (0.87–1.09)  1.00 (ref)  0.69 (0.37–1.30)  0.95 (0.53–1.68) | 0.680  0.252  0.856 | 1.02 (0.95–1.10)  1.00 (ref)  0.93 (0.56–1.54)  1.17 (0.75–1.83) | 0.617  0.774  0.488 |
| Multiglandular disease | 0.78 (0.38–1.62) | 0.506 | 0.82 (0.41–1.63) | 0.566 |

| **5b** | **Available cases** | | **Imputed data** | |
| --- | --- | --- | --- | --- |
| ***Women*** | **HR (CI 95%)** | ***p*** | **HR (CI 95%)** | ***p*** |
| Total calcium  continuous, mmol/L  2.17–  2.69–  2.82– | 1.43 (0.85–2.39)  1.00 (ref)  0.98 (0.75–1.28)  1.10 (0.85–1.44) | 0.177  0.879  0.464 | 1.82 (1.12–2.95)  1.00 (ref)  0.94 (0.72–1.23)  1.23 (0.95–1.59) | 0.015  0.659  0.122 |
| Adenoma weight  continuous, g  0.05–  0.38–  0.89– | 1.00 (0.95–1.06)  1.00 (ref)  1.13 (0.84–1.53)  0.95 (0.70–1.30) | 0.916  0.423  0.747 | 1.01 (0.96–1.06)  1.00 (ref)  1.17 (0.89–1.54)  0.99 (0.75–1.29) | 0.737  0.250  0.924 |
| Multiglandular disease | 1.35 (0.95–1.93) | 0.099 | 1.36 (0.97–1.92) | 0.078 |

| **5c** | **Available cases** | | **Imputed data** | |
| --- | --- | --- | --- | --- |
| ***Unmarried*** | **HR (CI 95%)** | ***p*** | **HR (CI 95%)** | ***p*** |
| Total calcium  continuous, mmol/L  2.17–  2.69–  2.82– | 1.75 (1.02–3.02)  1.00 (ref)  0.91 (0.66–1.26)  1.26 (0.93–1.70) | 0.043  0.559  0.130 | 1.77 (1.03–3.04)  1.00 (ref)  0.90 (0.66–1.24)  1.24 (0.92–1.67) | 0.039  0.518  0.162 |
| Adenoma weight  continuous, g  0.05–  0.38–  0.89– | 1.02 (0.96–1.09)  1.00 (ref)  1.15 (0.80–1.63)  0.98 (0.68–1.41) | 0.492  0.451  0.906 | 1.02 (0.96–1.08)  1.00 (ref)  1.10 (0.81–1.51)  0.99 (0.72–1.36) | 0.488  0.538  0.937 |
| Multiglandular disease | 1.35 (0.89–2.05) | 0.155 | 1.30 (0.86–1.97) | 0.217 |

| **5d** | **Available cases** | | **Imputed data** | |
| --- | --- | --- | --- | --- |
| ***Married*** | **HR (CI 95%)** | ***p*** | **HR (CI 95%)** | ***p*** |
| Total calcium  continuous, mmol/L  2.17–  2.69–  2.82– | 1.00 (0.48–2.07)  1.00 (ref)  1.27 (0.89–1.81)  1.11 (0.79–1.58) | 0.993  0.194  0.547 | 1.01 (0.49–2.09)  1.00 (ref)  1.21 (0.85–1.73)  1.16 (0.81–1.65) | 0.969  0.280  0.413 |
| Adenoma weight  continuous, g  0.05–  0.38–  0.89– | 0.96 (0.87–1.06)  1.00 (ref)  0.87 (0.57–1.34)  1.02 (0.68–1.53) | 0.465  0.540  0.923 | 0.98 (0.90–1.06)  1.00 (ref)  1.02 (0.71–1.49)  1.07 (0.75–1.51) | 0.606  0.900  0.709 |
| Multiglandular disease | 1.00 (0.61–1.66) | 0.992 | 0.99 (0.60–1.64) | 0.975 |

| **5e** | **Available cases** | | **Imputed data** | |
| --- | --- | --- | --- | --- |
| ***Income below median*** | **HR (CI 95%)** | ***p*** | **HR (CI 95%)** | ***p*** |
| Total calcium  continuous, mmol/L  2.17–  2.69–  2.82– | 1.56 (0.95–2.55)  1.00 (ref)  1.02 (0.78–1.34)  1.19 (0.92–1.54) | 0.078  0.873  0.177 | 1.98 (1.24–3.15)  1.00 (ref)  1.02 (0.79–1.33)  1.30 (1.01–1.67) | 0.004  0.856  0.041 |
| Adenoma weight  continuous, g  0.05–  0.38–  0.89– | 1.01 (0.95–1.06)  1.00 (ref)  1.03 (0.76–1.39)  1.03 (0.77–1.39) | 0.817  0.861  0.829 | 1.02 (0.98–1.06)  1.00 (ref)  1.10 (0.85–1.43)  1.09 (0.84–1.41) | 0.385  0.476  0.526 |
| Multiglandular disease | 1.23 (0.86–1.76) | 0.264 | 1.24 (0.87–1.75) | 0.233 |

| **5f** | **Available cases** | | **Imputed data** | |
| --- | --- | --- | --- | --- |
| ***Income above median*** | **HR (CI 95%)** | ***p*** | **HR (CI 95%)** | ***p*** |
| Total calcium  continuous, mmol/L  2.17–  2.69–  2.82– | 1.20 (0.47–3.10)  1.00 (ref)  1.11 (0.66–1.86)  1.20 (0.72–1.98) | 0.703  0.697  0.487 | 1.23 (0.50–3.06)  1.00 (ref)  1.00 (0.61–1.63)  1.29 (0.79–2.09) | 0.649  0.992  0.308 |
| Adenoma weight  continuous, g  0.05–  0.38–  0.89– | 0.96 (0.82–1.13)  1.00 (ref)  1.15 (0.61–2.16)  0.92 (0.50–1.72) | 0.621  0.674  0.805 | 0.98 (0.86–1.10)  1.00 (ref)  1.24 (0.73–2.12)  1.00 (0.60–1.66) | 0.706  0.431  0.998 |
| Multiglandular disease | 1.10 (0.55–2.23) | 0.785 | 1.13 (0.58–2.20) | 0.728 |

| **5g** | **Available cases** | | **Imputed data** | |
| --- | --- | --- | --- | --- |
| ***Income lowest quartile*** | **HR (CI 95%)** | ***p*** | **HR (CI 95%)** | ***p*** |
| Total calcium  continuous, mmol/L  2.17–  2.69–  2.82– | 2.04 (1.11–3.78)  1.00 (ref)  1.45 (0.99–2.14)  1.65 (1.14–2.38) | 0.022  0.059  0.008 | 2.59 (1.48–4.55)  1.00 (ref)  1.40 (0.96–2.03)  1.85 (1.30–2.65) | 0.001  0.081  0.001 |
| Adenoma weight  continuous, g  0.05–  0.38–  0.89– | 1.04 (0.98–1.11)  1.00 (ref)  0.94 (0.61–1.43)  1.09 (0.72–1.63) | 0.158  0.761  0.689 | 1.05 (1.00–1.11)  1.00 (ref)  1.13 (0.79–1.62)  1.23 (0.87–1.74) | 0.056  0.501  0.243 |
| Multiglandular disease | 1.08 (0.65–1.80) | 0.766 | 1.15 (0.72–1.85) | 0.560 |

| **5h** | **Available cases** | | **Imputed data** | |
| --- | --- | --- | --- | --- |
| ***Income above lowest quartile*** | **HR (CI 95%)** | ***p*** | **HR (CI 95%)** | ***p*** |
| Total calcium  continuous, mmol/L  2.17–  2.69–  2.82– | 1.13 (0.61–2.10)  1.00 (ref)  0.86 (0.63–1.17)  0.99 (0.74–1.33) | 0.704  0.332  0.952 | 1.26 (0.69–2.30)  1.00 (ref)  0.85 (0.64–1.15)  1.03 (0.77–1.38) | 0.451  0.301  0.849 |
| Adenoma weight  continuous, g  0.05–  0.38–  0.89– | 0.94 (0.85–1.04)  1.00 (ref)  1.11 (0.78–1.59)  0.95 (0.66–1.37) | 0.252  0.561  0.783 | 0.97 (0.90–1.05)  1.00 (ref)  1.09 (0.80–1.49)  0.96 (0.71–1.30) | 0.461  0.575  0.789 |
| Multiglandular disease | 1.28 (0.85–1.93) | 0.244 | 1.25 (0.83–1.87) | 0.289 |

| **5i** | **Available cases** | | **Imputed data** | |
| --- | --- | --- | --- | --- |
| ***Uniglandular disease*** | **HR (CI 95%)** | ***p*** | **HR (CI 95%)** | ***p*** |
| Total calcium  continuous, mmol/L  2.17–  2.69–  2.82– | 2.02 (1.23–3.31)  1.00 (ref)  1.14 (0.85–1.53)  1.39 (1.06–1.83) | 0.005  0.398  0.019 | 2.47 (1.55–3.92)  1.00 (ref)  1.09 (0.82–1.46)  1.52 (1.16–1.98) | <0.001  0.543  0.002 |
| Adenoma weight  continuous, g  0.05–  0.38–  0.89– | 1.00 (0.95–1.06)  1.00 (ref)  0.94 (0.69–1.26)  0.89 (0.66–1.20) | 0.872  0.667  0.444 | 1.02 (0.98–1.06)  1.00 (ref)  1.04 (0.78–1.39)  0.99 (0.75–1.32) | 0.411  0.772  0.960 |

Patients with multiglandular disease were too few to permit analysis.

| **5j** | **Available cases** | | **Imputed data** | |
| --- | --- | --- | --- | --- |
| ***Adenoma*** | **HR (CI 95%)** | ***p*** | **HR (CI 95%)** | ***p*** |
| Total calcium  continuous, mmol/L  2.17–  2.69–  2.82– | 1.67 (1.07–2.63)  1.00 (ref)  1.07 (0.83–1.40)  1.26 (0.99–1.62) | 0.025  0.592  0.062 | 1.99 (1.30–3.05)  1.00 (ref)  1.04 (0.81–1.34)  1.37 (1.07–1.74) | 0.002  0.767  0.011 |
| Adenoma weight  continuous, g  0.05–  0.38–  0.89– | 1.00 (0.95–1.05)  1.00 (ref)  1.04 (0.79–1.36)  1.01 (0.77–1.32) | 0.970  0.785  0.930 | 1.01 (0.97–1.06)  1.00 (ref)  1.10 (0.85–1.44)  1.06 (0.82–1.37) | 0.578  0.459  0.669 |
| Multiglandular disease | – | – | – | – |

Multiglandular disease was not possible to analyze due to the definition (>1 excised gland and primary histological diagnosis not adenoma). Patients with hyperplastic disease were too few to permit analysis.

### Figure S1. Kaplan-Meier survival estimates of patient status, national cohort. Log-rank test ns.

### Figure S2. Kaplan-Meier survival estimates of adenoma weight (g), national cohort. Log-rank test ns.
